# Antioncogenic Effects of Transient Receptor Potential Vanilloid 1 in the Progression of Transitional Urothelial Cancer of Human Bladder

**DOI:** 10.5402/2012/458238

**Published:** 2012-02-06

**Authors:** Giorgio Santoni, Sara Caprodossi, Valerio Farfariello, Sonia Liberati, Angela Gismondi, Consuelo Amantini

**Affiliations:** ^1^Section of Experimental Medicine, School of Pharmacy, University of Camerino, Madonna delle Carceri Street 9, 62032 Camerino, Italy; ^2^Department of Molecular Medicine, Sapienza University of Rome, Regina Elena Avenue 324, 00161 Rome, Italy

## Abstract

The progression of normal cells to a tumorigenic and metastatic state involves the accumulation of mutations in multiple key signaling proteins, encoded by oncogenes and tumor suppressor genes. Recently, members of the TRP channel family have been included in the oncogenic and tumor suppressor protein family. TRPM1, TRPM8, and TRPV6 are considered to be tumor suppressors and oncogenes in localized melanoma and prostate cancer, respectively. Herein, we focus our attention on the antioncogenic properties of TRPV1. Changes in TRPV1 expression occur during the development of transitional cell carcinoma (TCC) of human bladder. A progressive decrease in TRPV1 expression as the TCC stage increases triggers the development of a more aggressive gene phenotype and invasiveness. Finally, downregulation of TRPV1 represents a negative prognostic factor in TCC patients. The knowledge of the mechanism controlling TRPV1 expression might improve the diagnosis and new therapeutic strategies in bladder cancer.

## 1. Part 1

### 1.1. TRPV1: Structure and Function

The TRPV1 channel is predicted to have six transmembrane domains and a short, pore-forming hydrophobic stretch between the fifth and sixth transmembrane domains. It is activated by capsaicin [1], noxious heat (>43°C), low pH (5.2) [1–3], voltage [4, 5], various lipids [2, 6–11], and other pungent compounds such as zingerone, piperine, and those found in garlic and onion, such as allicin [12]. Similar to other six-transmembrane domain channels, TRPV1 probably forms a tetrameric quaternary structure [13], where each subunit contributes to the ion-conducting pore and the selectivity filter. Although all known TRP channels are cation selective, their permeability for different monovalent and divalent cations varies among their subtypes [14–16]. Ion permeation is controlled by allosteric interactions among the subunits and by an activation gate which, as for voltage-gated potassium channels, is most probably located in the innermost region of the S6 segment [17, 18]. In this regard TRPV1 channels also exhibit voltage-dependent behaviour [19].

Splice variants of the TRPV1 channel have been reported in several species. For example, the human TRPV1b splice variant, which lacks exon 7 corresponding to 60 aminoacids in the N-terminal region of the channel, can be found in DRG neurons and in the CNS [20]. It was first reported that TRPV1b could be activated by heat, but not by capsaicin or low pH [21]. However, in a more recent study it was demonstrated that this splice variant is unresponsive to vanilloid agonists, heat, and protons and can inhibit channel function by associating with canonical TRPV1, functioning as a dominant-negative variant, thus suggesting that it constitutes an endogenous TRPV1 modulator.

### 1.2. Expression of TRPV1 Channel in Normal Bladder Urothelium

Initially, TRPV1 expression was thought to be restricted to small diameter neurons within sensory ganglia [22]. Then, several studies have demonstrated the presence of TRPV1 also in nonneuronal cells and tissues such as rat thymocytes [23], human epidermal keratinocytes [24–26], smooth muscle [27], mast cells [25, 28], and hepatic stellate cells [29].

In the urinary bladder, the capsaicin-gated ion channel TRPV1 has been found to be expressed within afferent nerve terminals in rodent and in human species [30–32]. TRPV1-immunoreactive fibres were found in the mucosa and muscular layer of the entire urinary tract, among epithelial cells or closely apposed to smooth muscle cells. The first description of the expression of TRPV1 in rat urothelium, both at mRNA and protein levels, was by Birder group [30], that showed the expression of TRPV1 in basal and apical UCs lining the bladder lumen and in the interstitial cells. However, at present these data are in part questionable, since other studies have provided different evidence on the expression of TRPV1 in mouse, rat, and guinea pig UCs. Thus, Yamada et al. demonstrated barely detectable PCR product for TRPV1 in isolated mouse urothelium [33]; Everaerts et al. [34, 35] found negligible expression of TRPV1 mRNA, and they were unable to detect TRPV1 protein expression in mouse and rat UCs by using different specie-specific antibodies. By patch clamp electrophysiology, Xu et al. have demonstrated absence of capsaicin-evocated currents in urothelial cells from guinea pig [36]. Finally, Yu and Hill have recently failed to detect TRPV1 protein in mouse urothelium [37]. In this view, caution is necessary in the evaluation of the expression of TRPV1 protein in UCs from different species. The reasons for the confusion about urothelial expression include specie-specificity, low expression levels in some cases, presence of alternative splice variants of TRPV1, like TRPV1b [20, 38], poor specificity of antibody, the presence of nonurothelial cells in the urothelium, aspecific absorption of antibodies in the urothelium, culture conditions of naïve UCs, and so forth [37].

In human, Lazzeri et al. [39, 40] have exhaustively demonstrated the expression of TRPV1 mainly in the superficial urothelial cells, and recently its expression was confirmed by Charrua et al. [41]. Mechanical distention of the urothelium of isolated TRPV1 knockout (TRPV1^−/−^) mice bladders resulted in substantial decrease in ATP release [42], suggesting that TRPV1 has a functional role in normal bladder afferent mechanisms, for perception of mechanical and irritant stimuli [30, 42]. Exposure of UCs from TRPV1 knockout (TRPV1^−/−^) mice to resiniferatoxin (RTX) elicited none of the TRPV1-mediated responses, such as urothelial NO release [30].

TRPV1 appears necessary for normal bladder function, as TRPV1^−/−^ mice showed abnormal urodynamic responses, including increased frequency of nonvoiding contractions in the awake state and decreased frequency of reflex voiding contraction under anesthesia. TRPV1 appears to be required for bladder stretch detection, acting as both an initiator for urothelial ATP release and a mediator of hypotonically evoked ATP release.

TRPV1-expressing afferents lie in close proximity to, and sometimes traverse, the basal cell layer, and a “functional consortium” between urothelial and suburothelial TRPV1 has been proposed.

Patients with neurogenic detrusor overactivity (NDO) showed an increased expression of TRPV1, both in basal UCs and immunoreactive suburothelial nerve fibers [43, 44]; however, the contribution of urothelial *versus* neuronal TRPV1 has been not provided so far.

## 2. Part 2

### 2.1. TRP Channels and Tumorigenesis

The processes involved in the transformation of normal cells to tumorigenic cells and tumor progression are complex and only partly understood [45, 46]. The progression of cells from a normal, differentiated state to a tumorigenic, metastatic state involves the accumulation of mutations in multiple key signaling proteins, encoded by oncogenes and tumor suppressor genes, together with the evolution and clonal selection of more aggressive cell phenotypes. Some of the most important signaling pathways altered in tumorigenesis enhance cell proliferation and inhibit apoptosis. Ca^2+^ homeostasis controls these cellular processes, including proliferation, apoptosis, gene transcription, and angiogenesis [47].

TRP channels contribute to changes in intracellular Ca^2+^ concentrations, either by acting as Ca^2+^ entry pathways in the plasma membrane or via changes in membrane polarization, modulating the driving force for Ca^2+^ entry mediated by alternative pathways [48]. TRP proteins display an extraordinary diversity of functional properties and have profound effects on a variety of physiological and pathological conditions [48–50].

Approximately thirty TRPs have been identified to date and are classified in seven different families: TRPC (Canonical), TRPV (Vanilloid), TRPM (Melastatin), TRPML (Mucolipin), TRPP (Polycystin), TRPA (Ankyrin transmembrane protein) and TRPN (NomPC-like) [51].

In the recent years, TRP channels belonging to TRPV, TRPC, and TRPM families have been frequently associated with cancer growth and progression. Depending on the stage of cancer, either increased or decreased expression of TRP mRNA and protein levels have been reported. These changes may have cancer-promoting effects by increasing the expression of constitutively active TRP channels in the plasma membrane of cancer cells, thus enhancing Ca^2+^-dependent proliferative response. Alternatively, decreased expression of TRP channels may offer a survival advantage, such as resistance of cancer cells to apoptotic cell death.

At present, some of the TRP channels have been included in the tumor suppressor and oncogenic protein family. Indeed, in the TRPM family, TRPM1 has been suggested to be a tumor suppressor protein, and decrease in its expression appears to be a prognostic marker for metastasis in patients with localized malignant melanoma [52, 53]. Similarly, in the TRPM and TRPV family, TRPM8 and TRPV6 are considered oncogenes and their upregulated expression in prostate cancer may constitute new diagnostic markers for that disease [54–56].

In the next chapter we focalize our attention on the Antioncogenic properties of another member of TRPV channel family, TRPV1, by reporting published and unpublished findings supporting the protective role exerted by this receptor in the normal urothelium and the effects of its loss during the progression of transitional cell carcinoma (TCC) of human bladder, in the attempt to include the TRPV1 receptor into the anti-oncogene family.

### 2.2. Changes in TRPV1 Expression and Function during Neoplastic Transformation and Progression of TCC of Human Bladder

Urinary bladder cancer is the fifth most common neoplasm and the twelfth leading cause of cancer death. More than 90% of bladder carcinomas are TCC derived from the uroepithelium; about 6% to 8% are squamous cell carcinomas and 2% are adenocarcinomas. Stages Ta and Tis (in the urothelium) and stage T1 (in the lamina propria) are the nonmuscle-invasive stages. Most Ta tumors are low grade, and most do not progress to invade the bladder muscle. Stage T1 tumors are much more likely to become muscle invasive [57]. Alghout TCC of the urinary bladder is a chemosensitive neoplasm, metastatic disease is related with poor prognosis and short-term survival data. The emergence of novel biological agents offers the promise of improved outcomes, and many efforts are focused on the identification of new approaches to enhance chemotherapeutic efficacy [58, 59].

**Figure 1:**
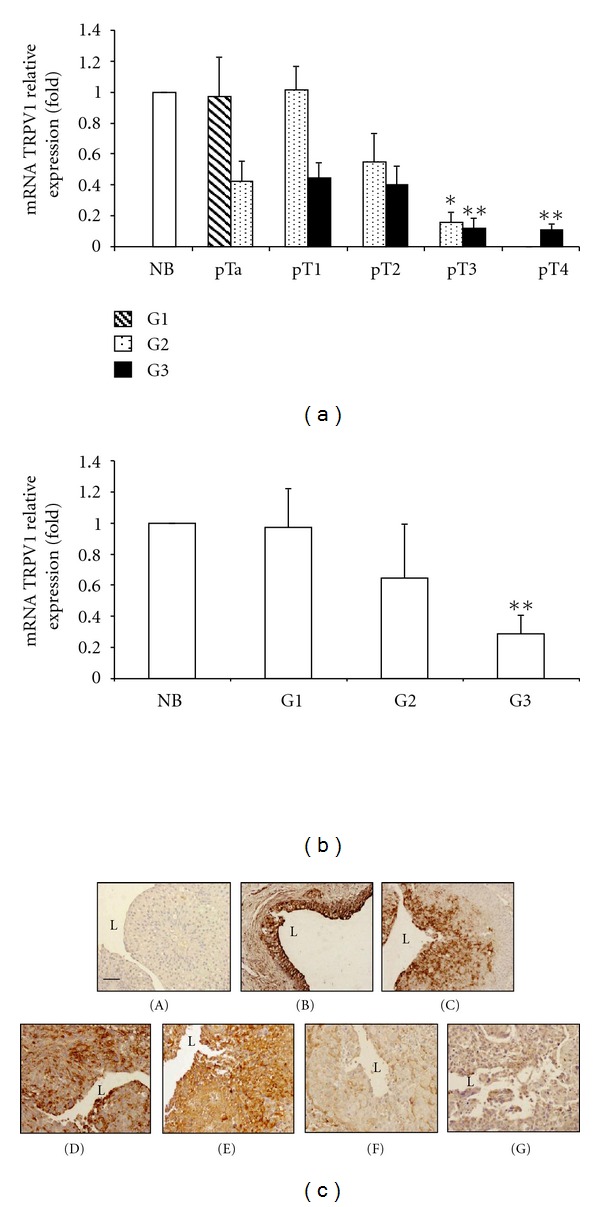
TRPV1 mRNA and protein expression in urothelial cancer (UC) tissues at different stages and grades. TRPV1 mRNA levels from UC tissues at different tumour stages (pTa–pT4) (a) and tumour grades (G1–G3) (b) were evaluated by quantitative real-time PCR. Results (mean ± standard deviation) were normalized for *β*-actin expression and TRPV1 levels were expressed as relative fold with respect to normal bladder (NB) tissues used as control (*0.01 ≤ *P* < 0.05; **P < 0.01, Kruskal-Wallis test). Sections from paraffin-embedded NB and UC tissues at different pathological stages (pTa–pT4) were immunostained with an anti-human TRPV1 antibody (c). (A) Omission of the primary antibody. (B) NB specimen. (C–G) UC specimens staged as pTa-pT4. Size bars: 25 *μ*m.L, lumen.

**Figure 2:**
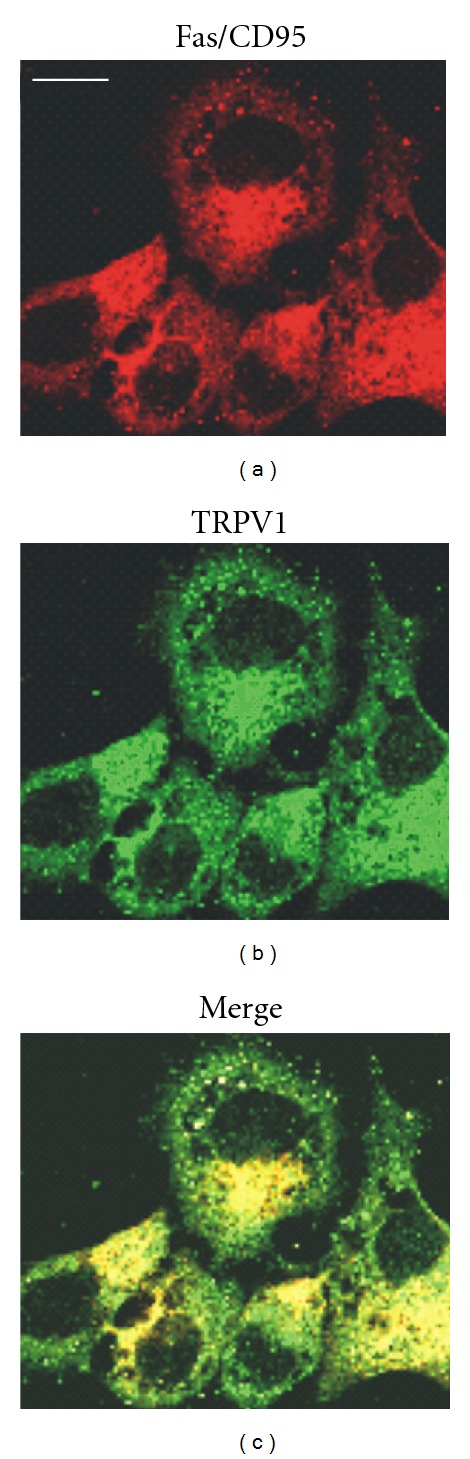
Capsaicin induces clustering and colocalization of Fas/CD95 and TRPV1 in RT4 UCCs. The immunocytochemical localization of Fas/CD95 and TRPV1 in UCCs treated with capsaicin was analyzed by confocal microscopy using an anti-Fas/CD95 mAb and a goat anti-TRPV1 Ab followed by respective secondary Abs. Merge panel indicates overlapping between Fas/CD95 and TRPV1 proteins. Bar = 10 *μ*m.

Changes in the TRPV1 expression can occur during the development of human urothelial cell carcinoma (UCC). Lazzeri and colleagues have demonstrated that TCCs show a progressive decrease in TRPV1 protein expression as the tumor stage increases [40]. In accordance with Lazzeri's data, we found [60] that TRPV1 was highly expressed at mRNA level in low-grade UCCs whereas its expression was strongly reduced in high-grade and stage invasive TCC (Figures 1(a) and 1(b)). Consistent with quantitative Real-time PCR data, a marked decrease or absence of TRPV1 labelling was found in UC specimens of high grades and stages as differentiation levels decreased (Figure 1(c)). Treatment of low-grade RT4 UCCs with the specific TRPV1 agonist, capsaicin at 100 *μ*M dose, induced a TRPV1-dependent G0/G1 cell cycle arrest and apoptosis. These events were associated with the transcription of proapoptotic genes including Fas/CD95, Bcl-2 and caspases, and the activation of the DNA damage response pathway. Moreover, stimulation of TRPV1 by capsaicin significantly increased Fas/CD95 protein expression and more importantly induced a TRPV1-dependent redistribution and clustering of Fas/CD95 that colocalized with the vanilloid receptor (Figure 2). These events suggest that Fas/CD95 ligand-independent TRPV1-mediated Fas/CD95 clustering results in death-inducing signaling complex formation and triggering of apoptotic signaling through both the extrinsic and intrinsic mitochondrial-dependent pathways [60]. In accordance with the Amantini group, previous evidence demonstrated that TRPV1 N-terminus binds to Fas-associated factor-1, a Fas/CD95-associated protein [61, 62], showing regulatory functions in TRPV1-dependent capsaicin-mediated apoptosis. Moreover, by the use of the specific ATM inhibitor KU55933, we found that capsaicin activates the ATM kinase involved in p53 Ser15, Ser20, and Ser392 phosphorylation. ATM activation is involved in Fas/CD95 upregulation and coclustering with TRPV1 as well as in UCCs growth and apoptosis.

In addition, recently findings indicated that capsaicin by triggering ROS production, mitochondrial membrane depolarization, also induced a TRPV1-dependent nonapoptotic cell death in T24 bladder cancer cells [63].

**Figure 3:**
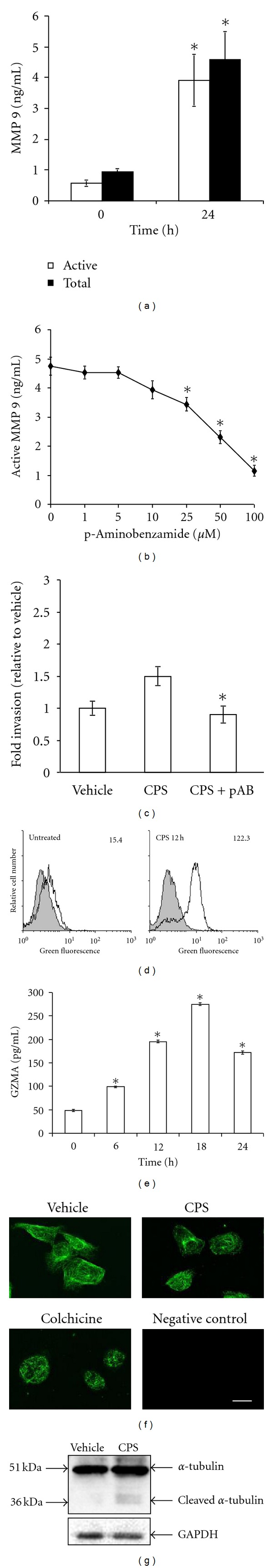
Capsaicin stimulates invasiveness of 5637 UCCs. Total and active MMP9 levels were evaluated in UCC lysates after capsaicin treatment by MMP9 activity assay. Statistical analysis was performed by comparing capsaicin with untreated cells, normalized for the relative vehicle, *P ≤ 0.01 (a). MMP9 levels were evaluated in UCCs treated with capsaicin alone or in combination with different doses of pAB. Statistical analysis was performed by comparing capsaicin-treated UCCs with capsaicin plus pAB-treated UCCs, *P ≤ 0.01 (b). Cell invasion was evaluated in UCCs treated with capsaicin in combination with pAB, by Matrigel invasion assay. Statistical analysis was performed by comparing capsaicin plus pAB-treated UCCs with capsaicin-treated UCCs, *P ≤ 0.01 (c). GZMA expression was evaluated in untreated and capasicin-treated UCCs by using a FITC-conjugated anti-human GZMA mAb and cytofluorimetric analysis. Numbers in the corner represent the mean fluorescence intensity. Gray areas represent the negative control (d). GZMA release was evaluated in the supernatant of untreated and capsaicin-treated UCCs by ELISA. Statistical analysis was performed by comparing capsaicin-treated with untreated UCCs, normalized with the relative vehicle treatments, *P ≤ 0.01 (e). Microtubule disassembly was evaluated by confocal microscopy using anti-human *α*-tubulin and FITC-conjugated goat Abs in vehicle or capsaicin-treated UCCs. Colchicine treatment was performed as positive control. Bar = l0 *μ*M (f). Total protein lysates from UCCs treated with capsaicin or vehicle were separated by electrophoresis and probed with anti-human *α*-tubulin followed by horseradish peroxidase-conjugated goat anti-mouse Abs (g).

Capsaicin has been found to exhibit either tumor-promoting or suppressing effects, in a receptor-dependent manner [64, 65]. We have recently provided evidence that capsaicin treatment induced a more aggressive gene phenotype and invasiveness in 5637 UCCs lacking TRPV1 receptor. Capsaicin treatment of UCCs induced upregulation of proangiogenetic (ANGPT1, ANGPT2, and VEGF), proinvasive and prometastatic genes (MMP1, MMP9, TIMP1, TIMP3, GZMA, NM23A, S100A) with a downregulation of apoptotic genes (Fas/CD95 and TNFRSF1A). Capsaicin increased the invasiveness of UCCs by triggering IGF-I release, granzyme A and MMP9 activation, *α*-tubulin disassembly, and cytoskeleton degradation (Figure 3). Finally, in 5637 UCCs transfected with the TRPV1 cDNA, we found an increase of capsaicin-mediated calcium level, growth inhibition, and apoptosis. Moreover, capsaicin-induced migration and MMP9 activation were reverted, suggesting that TRPV1 played an inhibitory role in UCC invasion and metastasis [65].

 In regard to the involvement of TRPV1 in capsaicin-induced antitumour effect *in vivo*, at present few data have been provided so far. *In vivo* experiments using capsaicin (5 mg/Kg body weight) injected once every 3 days during 4 weeks peritumorally in nude mice, showed that this vanilloid induced an antiproliferative effect and significantly slowed the growth of T24 bladder cancer xenografts [63]. Moreover, capsaicin at the concentration inducing apoptosis of MBT-2 murine bladder tumor cells, by reducing the level of reactive oxygen species and lipid peroxidation, enhances the anti-tumor effect of Bacillus Calmette-Guerin (BCG) in bladder cancer treatment [66]. Similarly, subcutaneous injection of capsaicin (5 mg/Kg body weight) in nude mice suppressed androgen-independent PC-3 prostate cancer cell growth in all tumors investigated and induced apoptosis of tumor cells [67]. Contradictory results were obtained by using subcutaneous injection of capsazepine (CPZ), a TRPV1 antagonist (5 mg/Kg body weight) in nude mice, that suppressed androgen-independent PC-3 prostate cancer cell growth [67]. Several reports indicate that CPZ is not the best TRPV1 antagonist, because sometimes it shows agonistic effects similar or better than capsaicin [2]. However, the *in vivo* agonistic effect of CPZ may be also evaluated on the view of the ability of the TRPV1 antagonists themselves to cause hyperthermia and consequently cell death in prostate cancer cells [68]. At present hyperthermia and intravesical therapy represent the gold-standard therapy in the management of TCCs [69, 70]. Long-term outcomes of randomized controlled trial have clearly demonstrated the superiority of the chemo- (mitomycin C-) hyperthermia regimen as compared to intravesical chemotherapy alone in terms of recurrence-free survival of bladder cancer patients.

Intravesical instillation of curcumin inhibits TCC cell implantation and growth in a murine superficial bladder tumor model [71]. Thus, it is rational and desirable the use of TRPV1 antagonists as adjuvant in combination to classic chemotherapy for bladder cancer treatment.

Finally, human TRPV1 expression has been found to be modulated in other tumors, and the Antioncogenic role of TRPV1 *in vivo* and *in vitro* has been further demonstrated.

Thus, TRPV1 has been found to exhibit tumor suppressive activity on skin carcinogenesis in mice because of its ability to down-regulate EGFR expression; conversely, loss of TRPV1 expression resulted in marked increase in papilloma development. TRPV1 by interacting with EGFR through its terminal cytosolic domain, facilitates Cbl-mediated EGFR ubiquitination and subsequently its degradation via the lysosomal pathway. In addition, ectopic TRPV1 expression in HEK293 cells resulted in decreased EGFR protein expression, and higher EGFR levels were observed in the skin of TRPV1-deficient mice (TRPV1^−/−^) as compared to wild-type control animals [72]. Moreover, a typical TRPV1 antagonist, AMG9810, promotes mouse skin tumor development via a significant increase in the expression level of EGFR and its downstream Akt/mTOR signalling pathway. Thus the application of this compound for classical pain relief might increase the risk of skin cancer [73]. Accordingly, curcumin inhibits both basal and EGF-induced growth and promotes autophagic cell death of 253JB-V and KU7 UCCs by down-regulating EGFR protein expression and inhibiting EGFR signalling [74]. By contrast, the cocarcinogenic effects of capsaicin on 12-O-tetradecanoylphorbol-13-acetate- (TPA-) promoted skin carcinogenesis *in vivo* is mediated through EGFR, but not by the TRPV1 receptor [75].

 Finally, TRPV1 mRNA and protein expression inversely correlated with glioma grading, with a marked loss of TRPV1 expression in the majority of grade IV glioblastoma tissues. TRPV1 activation by capsaicin induced apoptosis of U373MG glioma cells, and involved rise of Ca^2+^ influx, p38MAPK activation, mitochondrial permeability transmembrane pore opening and transmembrane potential dissipation, and caspase-3 activation [76]. In addition, TRPV1 expression has been also reported in human cervical cancer cell lines and tissues, and the endocannabinoid anandamide (AEA) induced TRPV1-dependent tumor cell apoptosis [77]. Finally, TRPV1 stimulation completely reverted the cannabidiol- (CBD-) mediated inhibitory effect on human cervical cancer cell invasion by blocking CBD-induced increase of TIMP-1 MMP inhibitor [78].

### 2.3. Diagnostic, Prognostic, and Therapeutic Role of TRPV1 in TCC of Human Bladder

Changes in TRP channel expression are associated with cancer development and metastasis. It has also been suggested that some TRP channels may serve as prognostic or diagnostic markers [31, 79]. Among the TRP superfamily, TRPV channels (TRPV1–6) are involved mainly in the regulation of growth and progression of genitourinary cancers. Thus, in prostatic adenocarcinoma, TRPV1 and TRPV6 are overexpressed with respect to healthy prostatic tissues, and their expression levels correlate strictly with Gleason score, pathological stage, extraprostat extension and tumor grades [80–82].

In this regard, we have recently assessed the role of TRPV1 mRNA downregulation as a negative prognostic factor in patients with bladder cancer [31].

**Figure 4:**
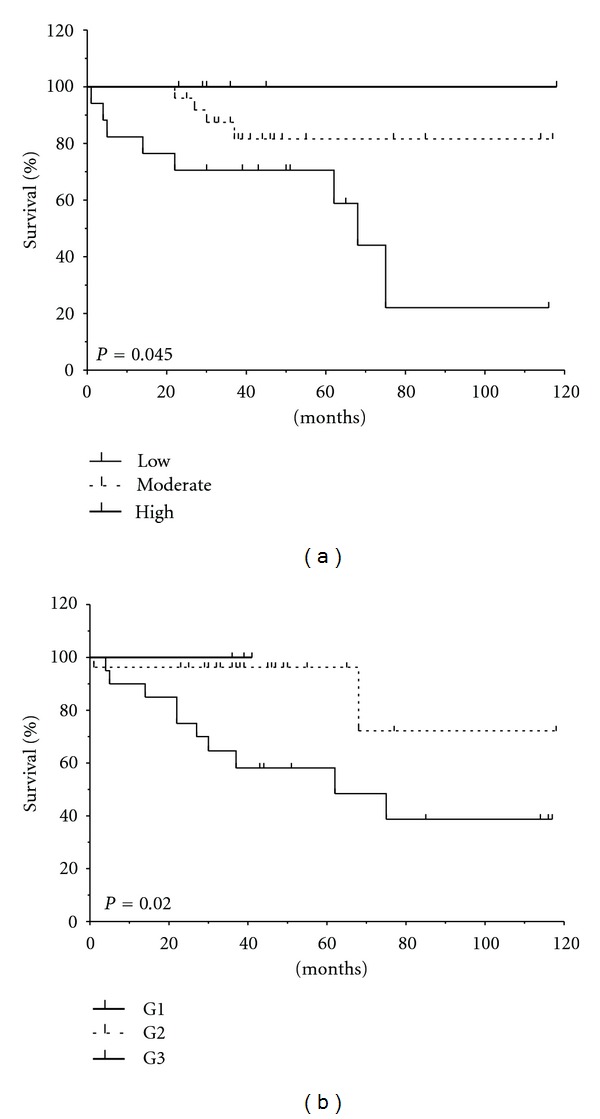
Decrease of TRPV1 mRNA expression is associated with a shorter survival of TCC patients. Kaplan-Meier survival analysis for patients without distant metastasis (M0) subgroup shows the association between survival and TRPV1 mRNA expression based on three categories: high, moderate, and low expression (a); survival and pathological grading divided into G1, G2, and G3 grades (b). Associated log-rank test *P* values were indicated for each analysis.

By univariate analysis, cumulative survival curves calculated according to the Kaplan-Meier method for the canonic prognostic parameters such as tumor grade and high stage (pT4), lymph nodes and distant diagnosed metastasis, reached significance. Notably, the reduction of TRPV1 mRNA expression was associated with a shorter survival of urothelial cancer patients (*P* = 0.008) and in a subgroup without distant diagnosed metastasis (*P* = 0.045) (Figure 4). In a multivariate Cox proportional hazards regression analysis, TRPV1 mRNA expression reached significance as an independent prognostic factor for survival considering all patients and the subgroup characterized by invasive stage. Taking into account that patients with metastasis generally have a poor prognosis [83], on a selected group with similar tumor grade and stage without distant diagnosed metastasis (M0) and lymph node positivity (N0), we found that TRPV1 could differentiate survival successfully as a valuable and independent molecular marker (Table 1). Thus, it is conceivable that the reduced expression of TRPV1 represent a mechanism by which TCCs evade anti-invasive and proapoptotic signals.

These findings may be particularly important in the stratification of urothelial cancer patients with higher risk of tumor progression for the choice of therapy options. Moreover, TRPV1 may be also useful to improve appropriate selection of postoperative follow-up protocols for individual patients.

 About 70% of TCC of human bladder are superficial at initial presentation. They are tumors confined to the mucosa (70%) or lamina propria (30%). Approximatively, 50 to 70% of these tumors recur with 10 to 30% showing grade and stage progression. TCCs with pT1G3 account for almost 10% of all TCC diagnosed, with respect to pT1G2-G1. They show a poorer prognosis with up to 50% progressing to muscle invasion with increased recurrence and progression rate, and invasiveness. In this regard, the significant reduction of TRPV1 expression we found in pT1G3 versus pT1G2 [31] that parallel that observed at protein level by Lazzeri et al. [40] may be particularly important in the evaluation of the stratification risk of recurrence and tumour progression of invasive versus non-invasive superficial TCC. Notably, since reduction of TRPV1 expression in TCC of human bladder was significantly associated with a shorter survival of urothelial cancer patients, the analysis of TRPV1 expression in pT1 G2–G3 TCC shows the presence of a risk group stratification: the first group of pT1G2 TCC patients showing a reduction of TRPV1 expression (25% of total) and the second group showing a marked reduction of TRPV1 expression (50% of total) [31].

In addition, it has been also found that expression of TRPV1 in TCC of human bladder is significantly reduced in nonmuscle-invasive versus muscle-invasive TCCs [31]. By analyzing 54 bladder tissue samples from nonmuscle-invasive (*n* = 28) and muscle-invasive (*n* = 26) TCC patients, we found a significative inverse correlation between TRPV1 mRNA expression and muscle invasiveness, suggesting that the negative prognostic value of reduction of TRPV1 mRNA in TCCs could be likely related to increased invasiveness of TCC in patients expressing lower TRPV1 level. Concordantly with these preliminary data, loss of TRPV1 in UCs was associated with a more aggressive gene phenotype and invasiveness in UCCs [65].

Moreover, Miao et al. have recently demonstrated in hepatocarcinoma patients that high TRPV1 expression is associated with increased disease-free survival [84].

In regard to treatment of TCC of human bladder, altogether we describe a novel connection between ATM DNA damage response and FasL-independent Fas-mediated intrinsic and extrinsic apoptotic pathways triggered by TRPV1 stimulation on TCCs. Many cancer cells acquire resistance to chemotherapeutic-induced cytotoxicity during tumor progression by decreasing their sensitivity to FasL/Fas-induced apoptosis [60]. Loss of Fas or FasL molecules, blocking the active FasL site by soluble sFas, seems to be induced in parallel to tumor progression. In addition, cell death induced by some cytotoxic drugs depend to an intact Fas system. Downregulation of Fas/FasL molecules as well as resistance to Fas-induced apoptosis has been reported in TCCs [71]. We found that capsaicin induces Fas upregulation both at transcriptional and translation levels, and more importantly FasL-independent TRPV1-dependent apoptosis, thereby bypassing some of the escape mechanisms triggered by TCCs. Similarly to FasL, the death ligand TRAIL has been found to induce apoptosis and sensitization of tumor cells to cytostatic or cytotoxic drugs [85]. In this regard, TRPV1 activation sensitized cancer cells to TNFR-mediated apoptosis [85]. Capsaicin has been found to upregulate DR5, a death receptor of TRAIL in UCCs [65].

### 2.4. Conclusion and Perspectives

Progress is required, not only in characterizing TRPV1 expression, activity, and distribution in TCCs, but also in addressing the feasibility of these TRP proteins as drug targets. This area of research is particularly significant, as the potential for the pharmacological modulation of channels is one of the key advantages over other targets.

The knowledge of the mechanism controlling TRPV1 expression would be of importance for a better understanding of UCC growth and progression. In the recent years urologists have developed a huge experience with intravesical instillations of vanilloids in the treatment of lower urinary tract (LUT) dysfunction. In particular, agonists of TRPV1 such as RTX, arvanil, olvanil have been considered as a new strategy to treat functional disorders of micturition reflex and pelvic-perineal pain in selected group of patients refractory to common therapies [43, 86–89]. The comprehension of the molecular mechanisms underlying their proapoptotic activity would be clinically relevant to extend the use of these agents also to the therapy of superficial urothelial malignancies. Thus, sustained expression of TRPV1 protein in low-grade superficial TCC and high stage-low grade muscle-invasive TCCs permits the utilize of specific TRPV1 agonists alone or in combination with chemotherapeutic drugs in the treatment of these tumors.

On the other hand, loss of TRPV1 during the progression of tumor with the acquisition of a more invasive phenotype stimulates studies on the mechanisms responsible to the expression of TRPV1 in TCC of human bladder. In this regard, the involvement of miRNA and E3-ligases in the control of TRPV1 mRNA and protein expression, respectively, and the study on the existence of a relationship between the expression of specific TRPV1 gene single nucleotide polymorphisms (SNPs) and splice variants and increased cancer risk of TCC of human bladder have been approached.

Further basic studies on the structure, *in vivo* expression and function of the TRPV1 channel must to be conduced to completely understand the role of TRPV1 as tumor suppressor gene in cancers of epithelial origins.

## Figures and Tables

**Table 1:** Multivariate Cox proportional hazard regression analysis of clinicopathological parameters and TRPV1 mRNA expression in relation to survival rates.

	Relative risk (95% CI)			
	All patients (*n* = 62)	pTa/T1 (*n* = 24)	pT2/T3/T4 (*n* = 38)	G2/G3 pT2/T3 N0M0 (*n* = 27)
TRPV1 (low/moderate/high)	0.26 (0.10–0.64) *P* = 0.004	0.61 (0.07–5.03) *P* = 0.65	0.30 (0.10–0.88) *P* = 0.030	0.19 (0.03–1.00) *P* = 0.05
Tumor grade (G1/G2/G3)	1.52 (0.74–3.12) *P* = 0.26	2.83 (0.29–27.44) *P* = 0.37	2.85 (0.81–10.12) *P* = 0.011	
Tumor grade (G2/G3)				5.65 (0.68–47.0) *P* = 0.11
Tumor stage (T2/T3)				0.43 (0.09–1.96) *P* = 0.28
